# Case Report: First use of dental bisphenol A-glycidyl methacrylate composite without UV light polymerization for repair of iatrogenic CSF leak following a frontal craniotomy plus tumor resection

**DOI:** 10.3389/fsurg.2024.1436361

**Published:** 2025-01-15

**Authors:** Haman Nassourou Oumarou, Ndome Toto Orlane

**Affiliations:** ^1^ Department of Surgery and Specialties, Faculty of Medicine and Biomedical Sciences, University of Yaounde 1, Yaounde, Cameroon; ^2^ Yaounde General Hospital, Yaounde, Cameroon; ^3^ Department of Surgery and Specialties, Faculty of Medicine and Pharmaceutical Sciences, University of Douala, Douala, Cameroon

**Keywords:** bis-GMA, CSF leak, reconstruction, osteo-meningeal breach, case report

## Abstract

**Objective:**

This study aimed to evaluate the efficacy and safety of bisphenol A-glycidyl methacrylate (bis-GMA) without UV light polymerization for the repair of refractory iatrogenic cerebrospinal fluid (CSF) leaks with large skull base defects.

**Background:**

CSF leakage remains a common complication after neurosurgical interventions with a substantial resultant impact on morbidity and increased healthcare costs. The management of refractory CSF leaks with large skull base defects remains challenging. Radiological investigations are highly contributive as they visualize the defect and assess the herniated content. Optimal treatment depends on the breach parameters and the consequent hernia. Surgery, when indicated, consists of exposure of the defect and its reconstruction using different grafts. The dental composite bis-GMA has been investigated and has shown effectiveness for the repair of anterior skull base defects. This is due to its compactible mechanical properties, long-term stability, and good osteo-integration. Hence, it presents a promising solution for refractory CSF leaks not responding to extradural endoscopic techniques.

**Case report:**

We describe the case of a 40-year-old female with persistent CSF rhinorrhea following a left frontal craniotomy performed 4 years before. A high-resolution cerebral CT scan and MRI revealed a bilateral fronto-ethmoidal osteo-meningeal breach and a hyperintense T2 signal in the ethmoidal sinus interrupting the hypo-intensity of the bone, respectively. In our patient, surgical treatment involved a bifrontal craniotomy and osteo-meningeal reconstruction with the use of bis-GMA without UV light polymerization. This reconstruction gave rigid structural support and watertight closure of the defect. Postoperatively, the CSF rhinorrhea ceased and there were no perceivable associated complications.

**Conclusion:**

Given the favorable outcome, the composite bis-GMA without UV light polymerization can be used as a reliable material for the repair of iatrogenic CSF leaks.

## Introduction

Cerebrospinal fluid (CSF) leakage is an egress of CSF via an osseous skull base dural defect with resultant direct communication of the extra-cranial space to the subarachnoid space (1). It is a common complication after neurosurgical interventions, especially skull base surgery, and is associated with significant morbidity and increased healthcare costs. Grotenhuis, in 2005, found an average cost difference of €17.412 for patients with postoperative CSF leakage compared to patients without CSF leakage (2). CSF leakage may lead to the development of a pseudomeningocele, wound healing problems, surgical site infections, meningitis, and pneumocephalus. It may arise from either congenital anomalies such as cephaloceles, a persistent craniopharyngeal canal, or cribriform plate defects or be acquired such as the post-traumatic or iatrogenic forms. Iatrogenic CSF leaks are now becoming common owing to the wide and routine use of complex skull base surgeries and functional endoscopic sinus surgery (3). However, little has been reported following non-basal skull surgeries with dural defects or with vault surgeries associated with frontal sinus defects. Delayed diagnosis and treatment of CSF leaks increase the risk of complications and hence, an accurate diagnosis is essential (4). High-resolution computed tomography provides excellent bone detail. Magnetic resonance imaging (MRI) accurately localizes the site of a CSF leak but has the limitation of poor spatial resolution and loss of bone detail. Thus, both CT and MRI serve as complementary modalities in CSF leak diagnoses (5).

Various surgical techniques have been employed for the repair of anterior skull base CSF leaks, especially those of iatrogenic origin, ranging from the endoscopic trans-nasal to the traditional open intracranial method. The endoscopic approach has gained popularity in recent years, most likely due to a high success rate, better field visualization, and few associated complications (6). The persistence of a CSF leak despite multiple surgeries may necessitate a transient or permanent CSF diversion. Despite using the previously described techniques, some cases, particularly those related to large skull base defects, may progress to refractory CSF leaks and necessitate further surgical exploration and intervention. In these circumstances, there is a paucity of literature on the choice of strategy. Due to the necessity of achieving a rigid and watertight repair of the skull base defect in order to minimize the risk of future CSF leaks, we opted to explore the efficacy of the biomedical graft bisphenol A-glycidyl methacrylate (bis-GMA). Sanus et al., in 2014, reported complete osteo-integration and realization of successful watertight closure of anterior skull base defects with the use of a bis-GMA-based allograft composite (7). Paul et al. also reported the successful repair of refractory CSF leaks in four patients in 2021 at the Yaounde General Hospital (6) with the use of bis-GMA polymerized with UV light. The utilization of a UV light source to polymerize the bis-GMA requires the introduction of a UV light source in the operating field, increasing the risk of postoperative surgical site infection. With this in mind, we explored the option of using bis-GMA without UV light polymerization of the composite. Thus, we share our experience of a successful repair of a CSF leak from an osteo-meningeal defect with the use of bis-GMA without UV light polymerization.

## Case presentation

**Figure 1:**
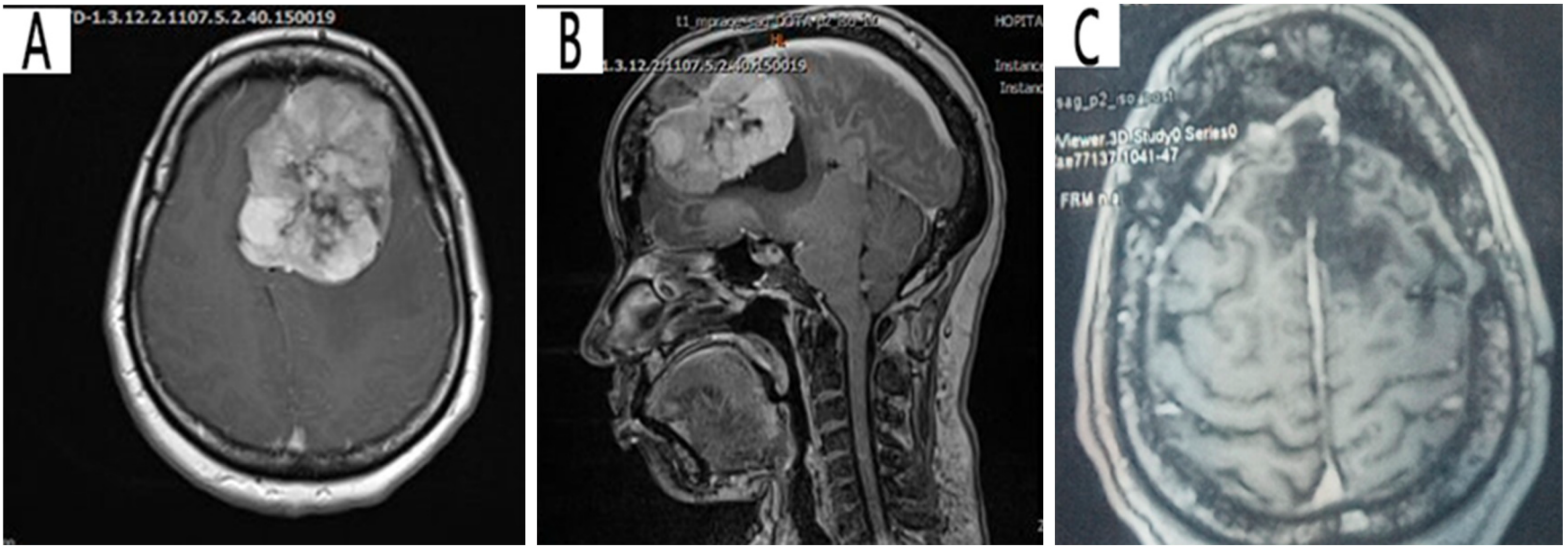
Pre-operative T1 cerebral MRI with contrast showing a left hyperintense frontal parasagittal mass with dural insertion: (**A**) axial view, (**B**) sagittal view **(C)** Postoperative T1 cerebral MRI showing the site of the tumor resection (porencephalic cavity).

**Figure 2:**
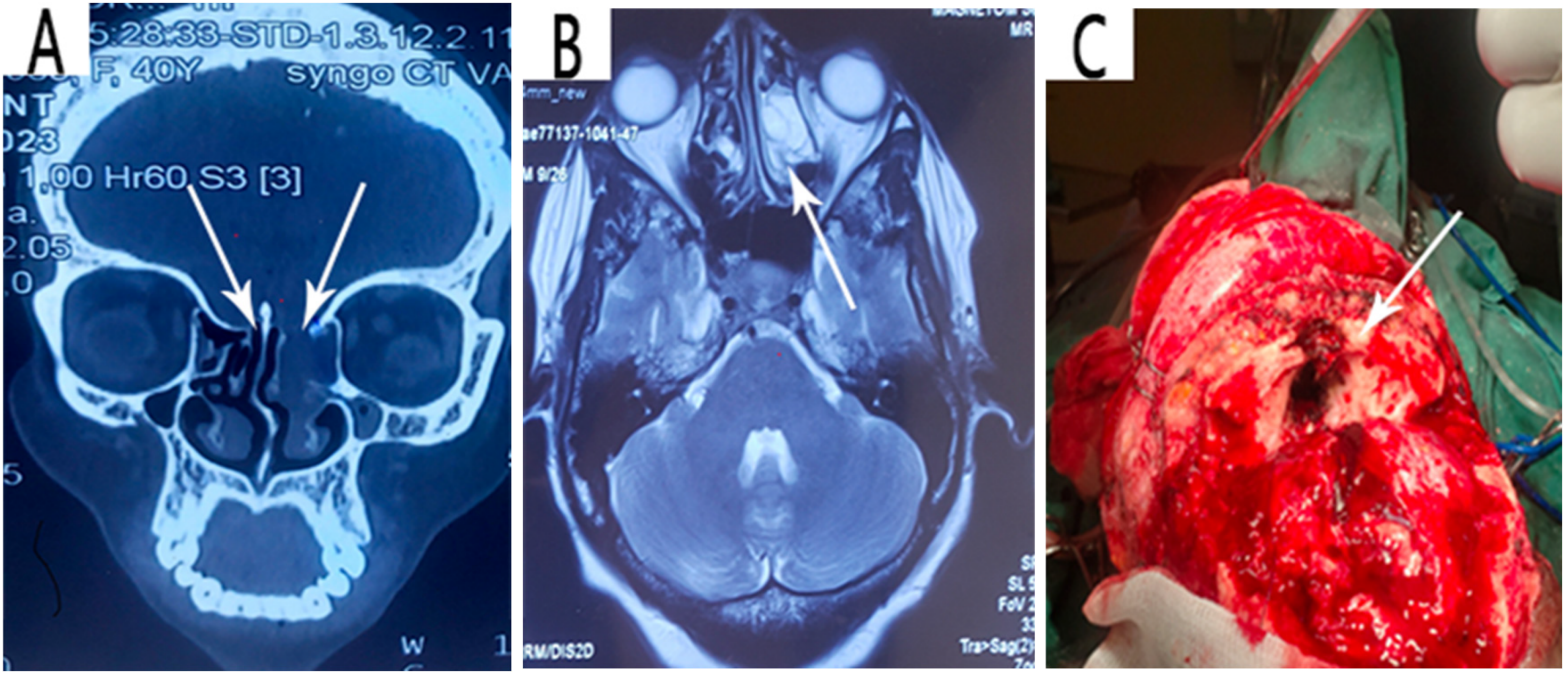
**(A)** Coronal view image of a high-resolution CT scan showing the bilateral fronto-ethmoidal osteo-meningeal breach at the anterior cranial base. **(B)** Axial T2 weighted MRI confirming CSF in the ethmoidal recess. **(C)** Intraoperative intracranial image showing the fronto-ethmoidal osteo-meningeal breach**.**

This is a case of a 40-year-old female artist who was resident in Yaounde, with a surgical history of left frontal craniotomy and complete tumor resection performed 4 years ago (Figure 1A–C). The immediate and short-term outcomes following her first surgery were unremarkable except for the presence of anosmia. She presented with late-onset clear, watery discharge from the nostrils when her head was tilted downward or when she sneezed. This was associated with a metallic taste but no history of headache, visual disturbances, meningitis, or seizures. She was non-hypertensive and non-diabetic with no personal or family history of tumors. A physical examination revealed clear serous fluid that exuded from the nostrils, mainly the left, exacerbated by posture (when the head was tilted down) with a positive reservoir sign and Valsalva maneuver. A cerebral CT scan revealed a bilateral fronto-ethmoidal osteo-meningeal breach (OMB) (Figure 2A). Cerebral MRI revealed a hyperintense T2 signal in the ethmoidal sinus interrupting the hypo-intensity of the bone (Figure 2B). A diagnosis of postoperative CSF fistula due to a fronto-ethmoidal osteo-meningeal breach was made. The patient was started on acetazolamide (Diamox 250 mg) and postural measures. Due to the persistent rhinorrhea despite the conservative medical therapy, an indication for more aggressive measures was posed.

**Figure 3:**
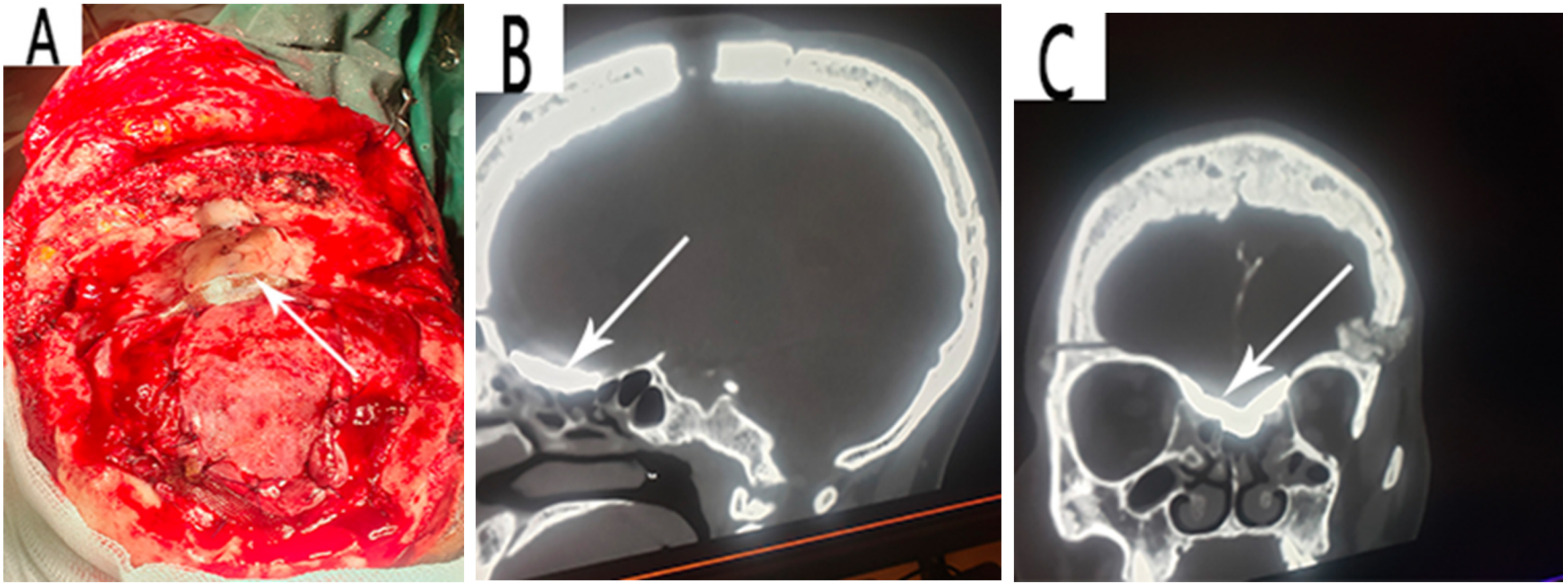
**(A)** Reconstruction of the defects with bis-GMA and pericranial flap. A follow-up CT scan showing visible fusion of the bis-GMA with the surrounding bone and complete sealing of the skull base bony defect **(B, C)**.

The patient was prepared for an operation with the goal of repairing the osteo-meningeal breach. An open surgery approach was utilized via a bicoronal incision and a bifrontal craniotomy and was tailored to be able to identify the extent of the osteo-meningeal breach (Figure 2C) and perform adequate reconstruction under general anesthesia. The patient's head was positioned to permit gravity to aid in the retraction of the frontal lobes. Following the craniotomy, the frontal lobes and their dura were retracted off the inner table of the cranial vault by gradual electrocoagulation to access the fronto-ethmoidal defect. A left frontal encephalocele, two sites of torn dura, and a bone defect on the posterior wall of the left frontal sinus were found perioperatively. The left frontal sinus mucosa was cauterized and blocked with bis-GMA, as was the ethmoidal defect (Figure 3A). The dural defects were repaired by direct suturing with the use of an autologous pericranial tissue graft reinforced by discontinuous simple suturing. Control hemostasis was achieved with the use of bipolar cauterization and surgicel. The postoperative evolution was uneventful, marked by the resolution of the CSF rhinorrhea but with the persistence of anosmia, and the patient was discharged 10 days after the surgery.

At the 1-month follow-up, there was no recurrence of the CSF rhinorrhea. The patient was able to perform her basic chores and there were no associated surgical complications or foreign body reactions. In addition, the patient had a normal neurological status except for anosmia. A follow-up CT scan revealed the visible fusion of the bis-GMA with the surrounding bone and complete sealing of the skull base bone defect (Figure 3B,C). At the 6-month follow-up, there was no recurrence of the CSF rhinorrhea and the patient had a normal neurological status. The patient's health-related quality of life evaluated by the SF-36 questionnaire gave average scores of 100 for role limitations due to emotional problems, social functioning, and bodily pain implying the patient had no problems with work or other daily activities as a result of emotional problems, performed normal social activities without interference due to physical or emotional problems, and presented no pain or limitations due to pain, respectively. The average scores of the other SF-36 scales are shown in Table 1.

**Table 1:** SF-36 health profile of the patient.

Scale	Label	Average score of recoded values
Physical functioning	PF	85
Role limitations due to physical health	RP	75
Role limitations due to emotional problems	RE	100
Vitality	VT	60
Mental health (emotional well-being)	MH	52
Social functioning	SF	100
Bodily pain	BP	100
General health	GH	50

## Discussion

Iatrogenic CSF leaks such as those following intracranial procedures can occur in patients of all ages. Coucke et al. reported an incidence rate of 3.8% for postoperative cerebrospinal fluid leakage after elective cranial surgery. They reported that skull base surgery had the highest rate of CSF leaks with 6.2% (8). Our case was that of supratentorial leakage from the anterior skull base underneath the site of initial craniotomy, hence the contributive role of the initial surgery to the appearance of the CSF leak cannot be minimized. CSF leaks from osteo-meningeal breaches can occur at any site although they are more likely to be found at fragile anatomical points. The most common defect location is the ethmoid cribriform plate (9), followed thereafter by the frontal and sphenoidal sinuses especially when hyperpneumatized (10). This was similar in our case as the patient had a bilateral fronto-ethmoidal osteo-meningeal breach.

A prompt diagnosis and the early treatment of CSF leakage minimizes the risk of severe complications. In patients presenting with clear fluid discharge, it is important to identify the cause. The etiology of CSF leaks can be post-traumatic, iatrogenic, spontaneous, or idiopathic, grouped into traumatic or non-traumatic causes (11). CSF leaks usually present as rhinorrhea that is exacerbated by the Valsalva maneuver without other associated rhinological signs (10). The nasal discharge may increase and become abundant with the Valsalva or with head-down maneuvers (Reservoir test). The halo sign presentation is a characteristic finding. A predisposing antecedent of prior facial trauma or nasal endoscopic or cranial base surgery should always be elucidated. The rupture of the natural fibrous scarring following minor trauma or increased intracranial pressure may account for the delayed presentation of liquorrhea (12), as was the case in our patient.

The differential diagnosis of CSF rhinorrhea is often challenging, especially in situations where the leaks are subtle. The confirmation of CSF rhinorrhea and localization of the causative defect may be diagnosed by CT, MRI cisternography, and MRI cisternography combined with single photon emission tomography or radioisotopic imaging. Although these methods allow for the estimation of the CSF leakage with high accuracy, they are expensive and invasive procedures. Therefore, biochemical methods are still used in the differentiation (11). Glucose concentration values greater than 0.3 g/L and protein concentrations up to 2 g/L are indicative of CSF (7). However, the analysis may be unreliable due to the need for large samples and contamination of the blood specimen increasing the false positive results rate up to 45%–75% (13, 14). Hence, there is a need for the more specific biochemical marker Beta-2 transferrin since it is found almost exclusively in CSF. Only a few other body fluids, such as the cochlear perilymph and the aqueous and vitreous humors in the eye, also contain it in low concentrations (1, 12). In our study, the diagnosis of CSF leak was clinical. This is because the diagnosis was obvious as our patient had an antecedent of a left frontal craniotomy and resection of a left frontal lobe mass that extended close to the anterior cranial base, hence biological exams were not essential.

The therapeutic choice of treatment of CSF leaks resulting from osteo-meningeal involvement must take into account the etiology, the anatomic site, the size of the defect, the severity of the CSF leak, and the timing and mode of presentation (15). A general rule of thumb is to always begin with the least invasive treatment and progressively advance to the more invasive treatments if necessary. Initially, conservative management measures are proposed when the CSF leak is minimal or the OMB is small in order to favor spontaneous scarring. These include measures such as bed rest and elevation of the head. Patients are encouraged to avoid coughing, sneezing, nose blowing, and any straining that would increase intracranial pressure. Stool softeners and laxatives may also be given if necessary to avoid straining during bowel movements and the use of drugs such as diuretics and carbonic anhydrase inhibitors to decrease CSF production. This simple approach is effective in 70%–85% of traumatic CSF leak cases (16, 17). Operative management is indicated when the CSF leak is abundant or refractory to medical treatment and when there are recurrent septic complications and multiple, bilateral, or large OMBs. In the case of our patient, the indication was a CSF leak refractory to medical treatment. Surgical procedures can be divided into intracranial and extra-cranial approaches. Endoscopic techniques have broadened the indications and improved the results associated with the extra-cranial repair of CSF leaks (16). With success rates as high as 90% reported, this approach is the most currently practiced due to its added advantage of aesthetic benefit and conclusive results (6). However, its application is limited to OMBs of the anterior skull base with no associated cerebral lesions. The intracranial approach (frontal craniotomy) is reserved for patients with defects that are not amenable to extra-cranial endoscopic techniques, patients with extensive skull base fractures, those with comminuted fractures with displaced fragments that require reduction, and those with fractures associated with intracranial hemorrhages or contusions that would ordinarily require a craniotomy for treatment (18). Intraoperative management includes the filling of small defects with bone dust and fibrin glue, or the use of split-thickness bone autografts, held in place with fibrin glue or one of the available microplate systems, when the defect is large.

Paul et al. in 2021 reported another intraoperative modality for OMBs with the use of a bis-GMA composite. The favorable results reported make this modality an option to be considered. The choice of our therapeutic surgical modality was based principally on the surgeon's familiarity, the results from prior usage of bis-GMA, and due to the fact that it is commercially available (6). Bis-GMA is a resin-based, non-resorbable composite derived from methacrylic acid and bisphenol A diglycidyl ether (19). Due to its biocompatibility and biomechanical properties conferred by its ability to form a cross-linked polymer with optimal curing and low volumetric shrinkage, it is widely used as a restorative dental composite and cement in dentistry (20). Its ability to offer rigid support has also been exploited in vertebral augmentation and craniofacial reconstruction and augmentation (21). The chemical composition of bis-GMA in the dental formula is approved by the Food and Drug Administration Authority (19). Of note, the use of the dental form of bis-GMA was adapted for the repair of skull base defects. Bis-GMA is a soft elastic paste, and its malleability allows it to be easily molded intra-operatively into different shapes within the surgical field to fit the osteo-dural breach. It has shown excellent biocompatibility and complete osteo-integration of the graft into the adjacent bones in animal skull base bone defects with the resultant achievement of a watertight seal of the dural and skull base defects without any complications reported in experimental models (20, 22). When the bis-GMA dental composite is exposed to UV light, it initiates a photochemical reaction that causes the monomers in the composite to cross-link and form a hardened, durable material (23). Thus, UV light polymerization ensures that the composite material is fully cured, leading to optimal mechanical properties and long-term stability. However, the need for a UV light source to initiate the polymerization process requires the introduction of more appliances in the operating theatre, thereby increasing the risk of surgical site contamination. In our patient, the use of an unpolymerized composite was justified by the utmost need to reduce the duration of the operation and the risk of contamination of the surgical site.

In addition to the bis-GMA dental composite, several dental amalgams exist. They are classified into two main types: low-copper dental amalgam and high-copper dental amalgam. Their use in the dental sector is backed up by the fact that they are easy to apply, have good durability, are economical, and have a bacteriostatic effect (24). Despite the diminishing popularity of amalgams in recent years due to their mercury content, and the wide availability of reliable composite materials, amalgams are still useful because they are excellent and versatile restorative materials. Furthermore, amalgams have greater longevity when compared to other direct restorative materials, such as composites. The properties, potential for use in skull base repair, and potential side effects of dental amalgams are presented in Table 2.

**Table 2:** Dental amalgams and their characteristic features.

Dental amalgams	Properties	Use in skull base repair	Potential side effects of brain interaction
Low-copper dental amalgams	Lower compressive strength (150–350 MPa), more corrosion and tarnish, more creep, greater incidence of marginal failure, slow setting reaction, require less speed and low energy amalgamation	Yes	Concern about mercury toxicity but no convincing evidence point to adverse health effects (24); no correlation between the presence of amalgam fillings and brain mercury level (25) Delayed hypersensitivity reactions possible; slight non-statistically significant increase between the presence of amalgam restorations and multiple sclerosis (26)
High-copper dental amalgams	High compressive strength (250–500 MPa), less corrosion and tarnish, less creep, fewer incidence of marginal failure, fast setting reaction, require high speed and energy amalgamation	Yes	

The short- and long-term outcomes of surgical repair of CSF leaks are often favorable. Endoscopic surgical repair of anterior skull base leaks is safe and effective with an average short-term failure rate of 9%. Long-term failure rates are low (27). The intracranial approach with the use of dental bis-GMA resin equally gave promising results (6, 7). In our patient, at the 6-month follow-up, there was no recurrence of CSF rhinorrhea and the patient had a normal neurological status. The patient's health-related quality of life evaluated almost 5 years after the first surgery by the SF-36 questionnaire was satisfactory in general.

## Conclusion

Surgery close to or involving the cranial base may be associated with occult iatrogenic osteo-meningeal breaches which may manifest in delayed CSF leaks, presenting most commonly as rhinorrhea. Medical imaging techniques, including high-resolution CT scans and MRI, are crucial for the diagnosis. Surgery is the primary treatment option, especially in refractory cases. Bis-GMA provides a promising solution for the repair of cranial CSF leaks via osteo-meningeal reconstruction due to its soft elastic nature that allows it to be molded intra-operatively, sealing both bone and dural defects, and its chemical property of complete osteo-integration. This prevents complications such as the significant risk of postoperative infection, postoperative graft failure, high rate of postoperative CSF leak, and foreign body reaction. The short- and long-term results of the usage of non-polymerized bis-GMA were promising. However, the advantage of minimal instrument manipulation in the operating theatre must be closely balanced with the risk of shrinkage, a potential complication of the incomplete curing of the composite that can lead to weaker and less durable restorations.

## Data Availability

The raw data supporting the conclusions of this article will be made available by the authors, without undue reservation.
